# Does one size fit all? The case for ethnic-specific standards of fetal growth

**DOI:** 10.1186/1471-2393-8-1

**Published:** 2008-01-08

**Authors:** William J Kierans, KS Joseph, Zhong-Cheng Luo, Robert Platt, Russell Wilkins, Michael S Kramer

**Affiliations:** 1 The British Columbia Vital Statistics Agency, Victoria, British Columbia, Canada; 2 The Department of Neonatal Pediatrics and the Perinatal Epidemiology Research Unit, Departments of Obstetrics and Gynecology and of Pediatrics, Dalhousie University Faculty of Medicine, Halifax, Nova Scotia, Canada; 3 Bureau-4986, Obstetrics and Gynecology, Sainte-Justine Hospital, University of Montreal, Montreal, Quebec, Canada; 4 The Departments of Pediatrics and of Epidemiology and Biostatistics, McGill University, Faculty of Medicine, Montreal, Quebec, Canada; 5 Health Analysis and Measurement Group, Statistics Canada, Ottawa, Ontario and Department of Epidemiology and Community Medicine, University of Ottawa, Ottawa, Ontario, Canada

## Abstract

**Background:**

Birth weight for gestational age is a widely-used proxy for fetal growth. Although the need for different standards for males and females is generally acknowledged, the physiologic vs pathologic nature of ethnic differences in fetal growth is hotly debated and remains unresolved.

**Methods:**

We used all stillbirth, live birth, and deterministically linked infant deaths in British Columbia from 1981 to 2000 to examine fetal growth and perinatal mortality in Chinese (n = 40,092), South Asian (n = 38,670), First Nations, i.e., North American Indian (n = 56,097), and other (n = 731,109) births. We used a new analytic approach based on total fetuses at risk to compare the four ethnic groups in perinatal mortality, mean birth weight, and "revealed" (< 10^th ^percentile) small-for-gestational age (SGA) among live births based on both a single standard and four ethnic-specific standards.

**Results:**

Despite their lower mean birth weights and higher SGA rates (when based on a single standard), Chinese and South Asian infants had lower perinatal mortality risks throughout gestation. The opposite pattern was observed for First Nations births: higher mean birth weights, lower revealed SGA rates, and higher perinatal mortality risks. When SGA was based on ethnic-specific standards, however, the pattern was concordant with that observed for perinatal mortality.

**Conclusion:**

The concordance of perinatal mortality and SGA rates when based on ethnic-specific standards, and their discordance when based on a single standard, strongly suggests that the observed ethnic differences in fetal growth are physiologic, rather than pathologic, and make a strong case for ethnic-specific standards.

## Background

Birth weight is the most commonly used measure of size; it is strongly associated with fetal, neonatal, and postneonatal mortality, infant and child morbidity, and long-term growth and performance [1]. Birth weight for gestational age is often used as an indirect measure of fetal growth, although true "growth" depends on serial increases in size over two or more time points during gestation. In the absence of valid and precise ultrasound or other noninvasive measures to assess true fetal growth *in utero*, birth weight for gestational age is used as an overall index of fetal growth from the time of conception to the moment of birth [2].

In using birth weight for gestational age for evaluating fetal growth in individual infants the question arises as to what is the appropriate standard to use. There is general agreement that sex-specific fetal growth standards are appropriate [1]. Female fetuses and newborn infants are smaller at any given gestational age than their male counterparts. Yet despite their smaller size, females are at lower risk for mortality and morbidity than males of the same gestational age.

Some investigators have also argued for ethnic-specific standards[3-6]. Within-country studies have shown that Chinese, Japanese, and (especially) South Asian infants are smaller for their gestational age[3,5-10], whereas North American Indian and North African infants are larger[11-16], than Caucasian infants in the same geographic setting, even after controlling for sociodemographic differences among the different ethnic groups. It has not been possible heretofore, however, to distinguish physiologic (i.e. normal or expected) from pathologic (i.e. adverse sequalae) effects in explaining these ethnic differences, even within the same population settings, and the case for ethnic-specific standards has not been widely accepted[1]. The recent development of a new analytic approach to pregnancy outcome based on fetuses at risk[17], rather than live births and/or stillbirths at a given gestational age, has enabled us to provide new insights into this issue. In this paper, we apply the new approach to the relatively large population of ethnic Chinese, South Asians, and First Nations (North American Indians), as well as Caucasians, in the Canadian province of British Columbia.

## Methods

The data used in this study are based on live birth and stillbirth registrations and notifications of birth received at the British Columbia Vital Statistics Agency (BCVSA) for births from January 1, 1981 to December 31, 2000. Infant death registration records from the Agency's death registry were linked and added to the birth records (including any infant deaths in 2001 that occurred to infants born in 2000). Links were deterministically based on birth registration number, which appears on the death record for infant deaths. In the case of infant deaths to former residents of British Columbia, inter-provincial agreements assured that the death record was available for linkage. The procedure resulted in a 98.9% linkage rate based on BCVSA infant death tables for 1981–2000 [18]. The confidentiality of BCVSA records was protected according to approved practices [18].

By British Columbia law, birth weight is recorded in hospital immediately after birth; <1% of deliveries occur out of hospital.

Since the early 1980s, ultrasound assessment is routinely performed in British Columbia early in the second trimester. The main source of BCVSA gestational age data prior to 1993 was the notice of birth completed by the attending physician (containing the gestational age as recorded by the physician, which is usually based on an early ultrasound estimate [19]), rather than the birth registration completed by the mother, and since 1993 the notice of birth has been the sole source. Furthermore, birth weights >4 SD at each week of gestation were identified during data analysis and corrected by accessing original documents, which were found for all but 4 cases; the latter were excluded from further analysis.

Analyses were restricted to singleton live births and stillbirths between 22 and 44 completed weeks of gestation with birth weights less than 7000 grams. Records where the weight, gestational age, or other study variables had missing or unknown values were excluded from the analysis.

For the purposes of this study, all births were allocated to one of four ethnic groups. Births were designated as Chinese if both the mother and father were born in the People's Republic of China, Hong Kong, Taiwan, Vietnam, or Singapore. Births were classified as South Asian if the mother and father were both born in Bangladesh, British India Ocean Territories, Sri Lanka, India, Nepal, or Pakistan. These locations were chosen in consultation with immigrant and cultural support organisations in British Columbia. Immigrants from these locations are considered to share Chinese or South Asian habits, culture, natality customs, and (most importantly) genetic heritage.

In recognition of current preferences, we use the term "First Nations" to refer to the third ethnic category. Status Indians are identified by means of a flag in the data set; their status is officially registered with the federal government and comprise the major part of the broad group of First Nations people in British Columbia, which also includes non-Status Indians, Inuit ("Eskimo"), and Metis. The major source for the data flag was the BCVSA statistical database of information extracted from the registration of births. Additional sources were the Indian Status Verification File provided by Health Canada's First Nations and Inuit Health Branch (which originates from the Department of Indian Affairs and Northern Development) and the Status Indian Entitlement files from the British Columbia Medical Services Plan. Using an extensive computer matching process, a birth was considered to be Status Indian if the mother was identified as a Status Indian in any of the three sources [20]. The term First Nations can be considered synonymous with "North American Indian" for most intents and purposes.

Births not included in any of the 3 ethnic groups specified above were allocated to the "Other" category and thus comprise Caucasian (primarily), mixed (mother-father), non-immigrant Chinese and South Asian, and Black ethnicities. Blacks are present in very small numbers in British Columbia and are not identified on the birth record.

The data file contained 865,968 records of singleton births including 4,456 stillbirths and 4,808 infant deaths at 22–44 weeks of gestation to residents of British Columbia. Chinese births totalled 40,092, South Asian births 38,670, and First Nations births 56,097, with the remaining 731,109 births in the "Other" group.

Gestational age-specific perinatal mortality was calculated as the number of perinatal deaths [stillbirths plus early neonatal (<7 days) deaths] at each completed week of gestational age, divided by the number of fetuses at risk at each gestation [17]. For example, perinatal mortality at 22 weeks gestation was calculated by dividing the number of perinatal deaths at 22 weeks by the number of live births plus stillbirths at 22 or more completed weeks of gestation, i.e., fetuses who delivered at 23, 24, 25, or more weeks of gestation were also at risk of live birth or stillbirth at 22 weeks.

Gestational age-specific patterns of fetal growth restriction were estimated using an indirect method based on the fetuses-at-risk approach. The number of "revealed" (see below) small-for-gestational-age (SGA) live births was determined for each group based on a birth weight < 10^th ^percentile for gestational age according to two different standards: (1) the current British Columbia live birth standard [18] and (2) an ethnic-specific live birth standard produced for each of the four ethnic groups under study by using the birth weight-for-gestational-age distributions specific to each group. "Revealed" SGA rates were then calculated by dividing the number of gestational age-specific SGA live births by the number of fetuses at risk at that gestation.

Because of the low absolute number of events (perinatal deaths, revealed SGA births) at early gestational ages for the Chinese, South Asian, and First Nations groups, we analyzed rates for these events as 2-week prospective risks. In other words, the rates were calculated as the number of events occurring during a given 2-week gestational period divided by the number of fetuses alive (and thus at risk for these events) at the beginning of that period. Neonatal deaths and stillbirths were analysed using the same method as perinatal deaths, with similar results (available on request).

We have previously shown that risks based on the number of fetuses at risk, rather than the number of total births, provides greater coherence between birth rates (and thus risks of early preterm birth), fetal growth restriction, and perinatal mortality [17,21-23]. One important consequence of using fetuses at risk rather than live births or total births as the denominator for calculating rates of gestational age-specific pregnancy outcomes is that perinatal mortality rates (and stillbirth and early neonatal mortality rates as well) rise with advancing gestational age. This may at first seem counter-intuitive, but conventional "rates" are actually ***ratios ***of deaths to live births or total births at a given gestational age. They are not true proportions, because the denominator does not include all subjects (unborn fetuses) at risk for the events denoted by the numerator; all living fetuses are at risk for stillbirth, live birth, and early neonatal death in the succeeding week. Neonatalogists are (appropriately) concerned with mortality among live-born births at a given gestational age, but neither the pregnant woman carrying a live fetus at a given gestational age nor her obstetrician, family physician, or midwife has any way of knowing whether or not her fetus will be born in the next week. From the woman's and her unborn fetus's perspective, the risk of stillbirth or live birth and early neonatal mortality in the succeeding week does indeed increase with advancing gestation, because the likelihood of birth (either a live birth or a stillbirth) rises as gestation advances [21].

Because SGA cannot be determined among unborn fetuses (i.e., those remaining in utero), and because the weight of stillbirths may underestimate the fetal weight at the (earlier) time of fetal death, we have developed a proxy measure, "revealed SGA," that provides a tip-of-the-iceberg indication of fetal growth restriction. The revealed SGA rate is the number of live-born SGA infants at a given gestational age divided by the number of fetuses at risk [17,21-23], where SGA is defined as a birth weight below the 10^th ^percentile birth at the given gestational age for this data set (i.e., an internal standard). Since the revealed SGA rate depends on both the birth rate and the SGA rate among live births, it is far below 10%, except in the last gestational age category (42+ weeks) when all remaining fetuses are born. It thus relates the number of live-born SGA infants to the number of fetuses at a given gestational age who were at risk for ***both ***SGA and birth during the subsequent week.

We used two different internal standards to define revealed SGA: (1) a single standard comprising all three study groups, and (2) a group-specific standard for each of the ethnic groups. We then graphically compared the patterns of gestational age-specific rates of live birth, revealed SGA, and perinatal death among the three study groups and compared the coherence of the patterns using the single vs group-specific SGA standards.

All statistical analyses were carried out using SAS-PC version 8.2. Specialized graphic output was produced using Microsoft Excel software Version 2002. Smoothing of the charts was accomplished using a 3^rd ^order polynomial calculated as the least squares fit through data points according to the following equation: y = b + c_1_x + c_2_x^2 ^+ c_3_x^3 ^where b and c are constants. Missing birth weight and gestational age values comprised <0.25% of total births in each group and were proportionally distributed across gestational age. Chinese and South Asians had the lowest percent missing, while First Nations was only slightly higher than Others. BCVSA makes a particular effort to include birth weight and gestational age values on all records. If either measure is not recorded on the notice of birth, the source is contacted before the record is processed.

## Results

**Figure 1:**
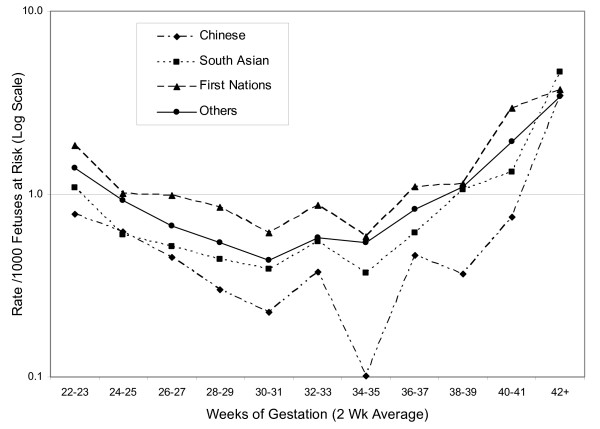
Perinatal mortality per 1000 fetuses at risk in four ethnic groups.

The LBW rate (for total births) was 4.2% among Chinese, 6.3% among South Asian, 5.6% among First Nations, and 4.4% among Other births. The preterm birth (<37 completed weeks) rate was 5.3% among Chinese, 6.8% among South Asian, 9.3% among First Nations, and 5.6% among Other births. Table 1 shows the number of principal study outcome events and fetuses at risk for each ethnic group at 22 to 42+ completed weeks of gestation. The low numbers of outcome events for Chinese, South Asian, and First Nations births are apparent, supporting the need for analysis by 2-week prospective intervals. As shown in Figure 1, the First Nations group showed the highest perinatal mortality rates at all gestational ages (except at 42+ weeks), whereas the Chinese and South Asian groups consistently showed the lowest rates.

**Table 1:** Gestational Age-Specific Outcomes for Four Study Groups

	Chinese						South Asian						First Nations						Other					
	SB	LB	END	SGA_1_	SGA_4_	FAR	SB	LB	END	SGA_1_	SGA_4_	FAR	SB	LB	END	SGA_1_	SGA_4_	FAR	SB	LB	END	SGA_1_	SGA_4_	FAR

22	13	6	6	1	0	40092	12	12	11	2	1	38670	35	24	23	0	2	56097	357	193	180	17	17	731109
23	8	6	4	0	1	40073	13	9	6	0	1	38646	27	26	19	2	2	56038	257	260	222	21	21	730559
24	8	11	5	2	1	40059	1	19	9	1	1	38624	18	28	16	1	2	55985	237	356	191	29	23	730042
25	6	16	6	0	2	40040	7	19	6	1	1	38604	11	39	11	5	4	55939	130	334	118	21	21	729449
26	2	14	4	2	1	40018	3	33	4	5	4	38578	11	55	22	5	4	55889	142	487	100	39	39	728985
27	4	19	2	2	2	40002	3	33	7	9	3	38542	13	74	10	3	6	55823	102	478	63	44	44	728356
28	4	22	2	2	2	39979	2	40	5	5	4	38506	14	98	10	5	9	55736	153	667	79	63	63	727776
29	4	35	0	3	4	39953	7	43	1	5	4	38464	7	98	6	3	8	55624	95	656	43	69	63	726956
30	3	44	1	2	1	39914	4	72	3	8	7	38414	13	140	8	6	11	55519	141	910	37	93	89	726205
31	2	57	1	4	4	39867	2	61	0	10	7	38338	7	147	7	9	14	55366	90	1033	51	99	97	725154
32	3	89	5	9	9	39808	6	110	3	20	11	38275	17	277	11	12	26	55212	169	1905	66	168	168	724031
33	5	129	2	13	11	39716	7	140	5	25	14	38159	17	332	3	16	28	54918	128	2367	56	190	204	721957
34	1	223	1	11	12	39582	5	288	1	32	24	38012	10	588	4	27	50	54569	154	4423	51	375	360	719462
35	2	353	0	19	24	39358	4	410	3	50	31	37719	10	781	8	40	60	53971	131	7003	54	572	572	714885
36	5	1,005	3	79	86	39,003	9	1,210	2	131	93	37,305	25	2205	6	111	187	53180	215	16468	92	1379	1304	707751
37	7	2,378	3	173	205	37,993	9	2,168	2	255	175	36,086	25	3566	2	213	326	50950	189	31293	90	2665	2558	691068
38	5	7,642	2	749	699	35,608	7	6,180	6	820	566	33,909	22	8669	6	556	795	47359	276	87154	113	7661	7661	659586
39	5	10,626	1	1351	1040	27,961	17	9,480	5	1444	891	27,722	18	10818	8	756	1034	38668	232	139163	103	12752	13250	572156
40	7	12,278	1	1847	1196	17,330	14	12,543	2	2211	1232	18,225	42	20544	20	1529	2016	27832	379	267794	197	25113	26383	432761
41	3	4,177	2	698	407	5,045	7	4,589	1	823	452	5,668	14	5611	6	421	546	7246	183	119539	78	11110	11822	164588
42+	0	865	3	147	85	865	2	1,070	2	232	103	1,072	6	1615	0	163	176	1621	96	44770	57	4200	4315	44866

Sum	97	39995	54	5114	3792		141	38529	84	6089	3625		362	55735	206	3883	5306		3856	727253	2041	66680	69074	

SB = stillbirth; LB = live births; END = early neonatal death; SGA_1 _= small-for-gestational age based on single standard; SGA_4 _= small-for-gestational age based on 4 ethnic-specific standards;

FAR = fetuses at risk

Data source: British Columbia Vital Statistics Agency

**Figure 2:**
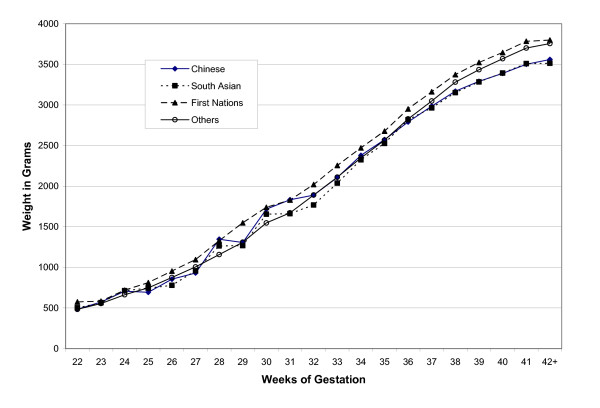
Mean birth weight for gestational age in four ethnic groups.

In contrast to the perinatal mortality curves, mean birth weights were consistently highest in First Nations births, whereas those for Chinese and South Asian births progressively lagged behind those of Other births after 35–36 weeks (Figure 2). These differences in trajectories of mean birth weight for gestational age culminated in differences of almost 200 grams at 42+ weeks.

**Figure 3:**
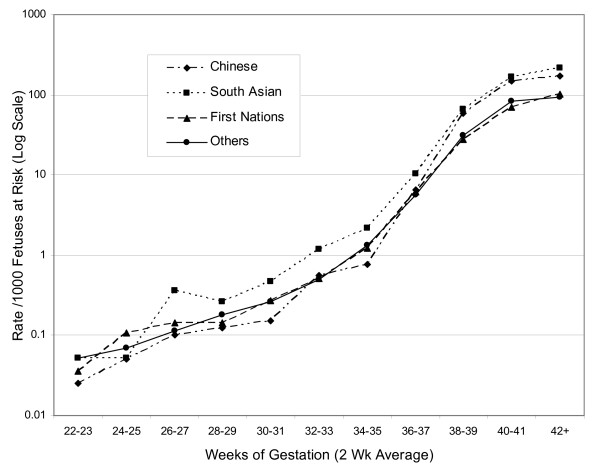
Revealed SGA rate per 1000 fetuses at risk in four ethnic groups, based on a single standard.

As shown in Figure 3, revealed SGA rates based on the single British Columbia standard were consistently highest for South Asian births at all gestational ages. Revealed SGA rates among the Chinese rose relative to those for First Nations and Others after 36 weeks and became comparable to those of South Asians at and after term. First Nations rates remained similar to those of Other births at most gestations.

**Figure 4:**
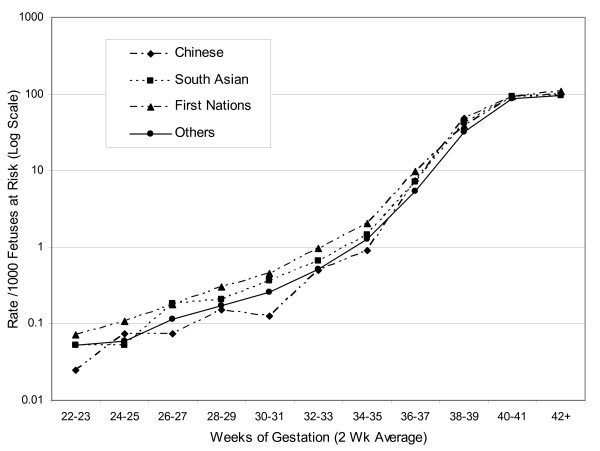
Revealed SGA rate per 1000 fetuses at risk in four ethnic groups, based on 4 ethnic-specific standards.

Revealed SGA rates based on ethnic-specific fetal growth standards (Figure 4), however, showed a very different pattern, one that was far more consistent with the perinatal mortality differences shown in Figure 1. Differences among ethnic groups near and after term were much smaller than those based on the single British Columbia standard. Rates for First Nations births were highest before term. Revealed SGA rates among South Asian births were slightly higher than those of the Other group throughout most of gestation. Chinese births showed relatively low rates prior to 36 weeks, after which they progressively rose to reach or exceed those of the three other ethnic groups.

Table 2 shows that perinatal mortality rates among Chinese were substantially lower than perinatal mortality rates among Others at 35 and 40 weeks gestation and not significantly different at 41 weeks. This pattern of mortality was not congruent with patterns of small-for-gestational age as defined using a single standard. Revealed SGA rates were lower than those among the Others at 35 weeks but substantially and significantly higher at 40 and 41 weeks gestation (rate ratio 1.84 and 2.05 at 40 and 41 weeks, respectively). Revealed SGA pattern based on ethnic-specific standards were more congruent with patterns of gestational age-specific perinatal mortality, with rates being significantly lower at 35 weeks gestation and only slightly higher at 40 and 41 weeks gestation (rate ratio 1.13 and 1.12 at 40 and 41 weeks, respectively). Among South Asians, perinatal mortality rate were similar to those among Others. However, patterns of revealed SGA were very different from patterns of perinatal mortality when SGA was defined using a single standard (rate ratio 2.09 and 2.15 at 40 and 41 weeks, respectively). Revealed SGA patterns based on an ethnic-specific definition of SGA resulted in revealed SGA rates more congruent with patterns of perinatal mortality (Table 2). Rates of perinatal mortality and revealed SGA among First Nations subjects were also incongruent when SGA was defined using a single standard and more consistent when SGA was defined using an ethnic-specific standard. Whereas perinatal mortality rates were higher among First Nations compared with others, revealed SGA rates based on a single standard were the same or lower. When an ethnic based standard was used to define SGA, however, revealed SGA rates were higher among First Nations than among Others (rate ratio 1.39, 1.19 and 1.05 at 35, 40 and 41 weeks, respectively).

**Table 2:** Rates of perinatal mortality and revealed small-for-gestational-age at 35 weeks, 40 weeks and 41 weeks by ethnicity

Ethnic group (gestational age)	Perinatal mortality			Revealed SGA rate (single standard)			Revealed SGA rate (ethnic-specific std.)		
	Rate	Rate ratio	95% CI	Rate	Rate ratio	95% CI	Rate	Rate ratio	95% CI

35 weeks									
Chinese	0.05	0.20	0.05–0.79	0.5	0.60	0.38–0.95	0.6	0.76	0.51–1.15
South Asian	0.19	0.72	0.34–1.53	1.3	1.66	1.24–2.21	0.8	1.03	0.72–1.47
First Nations	0.33	1.29	0.79–2.09	0.7	0.93	0.67–1.28	1.1	1.39	1.07–1.81
Other	0.26	1.00	-	0.8	1.00	-	0.8	1.00	-
40 weeks									
Chinese	0.46	0.35	0.17–0.70	106.6	1.84	1.76–1.92	69.0	1.13	1.07–1.20
South Asian	0.88	0.66	0.40–1.08	121.3	2.09	2.01–2.18	67.6	1.11	1.05–1.17
First Nations	2.23	1.67	1.29–2.17	54.9	0.95	0.90–1.00	72.4	1.19	1.14–1.24
Other	1.33	1.00	-	58.0	1.00	-	61.0	1.00	-
41 weeks									
Chinese	0.99	0.62	0.26–1.51	138.4	2.05	1.91–2.20	80.7	1.12	1.02–1.23
South Asian	1.41	0.89	0.44–1.80	145.2	2.15	2.01–2.30	79.7	1.11	1.01–1.21
First Nations	2.76	1.74	1.11–2.74	58.1	0.86	0.78–0.95	75.4	1.05	0.99–1.14
Other	1.59	1.00	-	67.5	1.00	-	71.8	1.00	-

Data source: British Columbia Vital Statistics Agency

These data spanned a 20-year period and temporal trends in immigration and mortality might conceivably bias overall outcomes. To address this possibility, we re-analysed the birth and perinatal mortality data within 5-year periods from 1981 to 2000. A slight increase was observed in the proportion of births to First Nations and a slight decrease in the proportion born to the Other group, but perinatal mortality rates declined for both groups over the 20-year period. The proportion of Chinese and South Asian births increased substantially in the 1990s, while perinatal mortality rates rose slightly, although fluctuations due to low numbers resulted in some instability in the trends. A general convergence of mortality rates occurred, although the groups generally maintained their relative positions.

## Discussion

We found lower mean birth weights and higher rates of revealed SGA among ethnic Chinese and South Asian births compared with other ethnic groups when the classification of SGA was based on a single British Columbia standard of birth weight for gestational age. First Nations births, on the other hand, had higher mean birth weights, and slightly lower revealed SGA rates near and after term, compared with other ethnic groups. These results are similar to those reported by previous investigators[3,5-10,13-15] and thus in themselves are not surprising. What is new is our finding that Chinese and South Asian fetuses are at lower risk of perinatal death throughout gestation despite their smaller size. Conversely, First Nations fetuses are at uniformly higher perinatal death risk despite their larger size. In other words, fetal growth and perinatal mortality show discordant results among the four ethnic groups under study, at least when fetal growth is classified using a single standard of birth weight for gestational age.

When ethnic-specific standards are used to define SGA, however, revealed SGA rates and perinatal mortality rates become far more concordant. To the extent that SGA prevalence among live-born infants reflects an adverse intrauterine environment [21], it should indeed be reflected by rates of perinatal death. We have reported precisely such a pattern in comparisons of births to primiparous vs multiparous mothers, smokers vs nonsmokers, twins vs singletons, and U.S. Blacks vs Whites [17,22]. The ethnic-specific standards for SGA yield revealed SGA rates that are concordant with the corresponding perinatal mortality rates (except for a slightly higher rate in South Asians than in the Other group), whereas the single standard results in discordance between the rates. In our view, this evidence justifies the consideration of ethnic-specific standards of birth weight for gestational age, at least for Chinese, South Asian, and North American Indian ethnicities. The lower perinatal mortality rates in Chinese and South Asian pregnancies despite their smaller size parallels the well-recognized pattern in female vs male fetuses, for whom sex-specific standards of birth weight for gestational age have been advocated for some time[1,22]. We have previously shown, however, that the fetuses-at-risk approach does not support the use of separate standards in U.S. Blacks vs Whites[23].

Our finding that gestational age-specific patterns of revealed SGA cohere better with gestational age-specfic perinatal mortality when ethnic-specific standards were used suggests that ethnic-specific birth weight for gestational age represent a physiologic (i.e. normal or expected) rather than a pathologic process. If differences in fetal growth (as reflected by GA-specific mean birth weights and revealed SGA rates) were truly pathologic, rather than physiologic, we would expect patterns that were more coherent with those observed for perinatal mortality when the definition of SGA was based on a single population standard, rather than ethnic-specific standards.

The fact that Chinese and South Asian births in British Columbia occur in immigrants (by definition--see Methods) raises the question of a "healthy migrant" bias and the generalizability of our findings to ethnic Chinese and South Asian births in China, South Asia, and elsewhere. Although it is possible that Chinese and South Asian parents who succeeded in immigrating to Canada are better educated and more socio-economically advantaged than those who remained in their countries of origin or those in the indigenous (non-immigrant) British Columbia population, the neighbourhood income distributions of the immigrant parents in our sample were lower than the income distribution in the province overall [18]. All else being equal, therefore, one should expect a higher gestational age-specific perinatal mortality risk relative to the British Columbia majority (Other) ethnic group. We found the opposite, however. Moreover, the lower mean birth weights and higher revealed SGA rates (when based on a single standard) among Chinese and South Asian births are consistent in direction, if not in magnitude, with those published from other settings[3,5-10]. The fact that our study is population-based, rather than hospital-based, makes selection bias ***within ***the British Columbian population of births over a 20-year period a highly unlikely explanation for our findings. We are unaware of any selection factors that would simultaneously lead to smaller fetuses/infants and lower perinatal mortality.

Our mortality rates in the South Asian group were lower than those in the Other group which contradicts the findings in some European studies [24,25]. However, population-based studies [26,27] in the United States have reported lower neonatal and postneonatal mortality rates among newborns of "Asian-Indian" immigrant mothers compared to indigenous white mothers. The difference between the two continents could be due to varying demographic, socioeconomic, and/or environmental characteristics of the underlying immigrant source populations. Those characteristics are not available in other reports but our South Asian mothers have slightly lower socioeconomic status compared to Other mothers (see above), are mostly from the Punjab area of India (Sikhs), and almost wholly non-smoking urban dwellers with Canada's universal access to medical care.

We have previously argued for separate analysis of stillbirths and early neonatal deaths and against their combination as "perinatal deaths" when gestational age-specific perinatal mortality risk is based on using total births (stillbirths plus live births) as the denominator for calculating the risk [23]. Using total births as the denominator is incorrect whenever the numerator (number of outcome events) includes stillbirths, since fetuses who remained *in utero *during the risk period (e.g., a given week of gestation) are at risk of stillbirth during the period but are not counted in the denominator at risk. With fetuses at risk in the denominator, however, the combination of stillbirths and early neonatal deaths as perinatal deaths is no longer problematic. Indeed, we have carried out separate analyses of stillbirths and neonatal deaths based on the fetuses-at-risk approach, and the results (available on request) are entirely consistent with those presented here for perinatal deaths.

While factors other than ethnicity (including altitude, maternal size, parity, smoking, parental social position) can affect fetal growth and could confound the ethnic differences we observed; numerous previous studies[3,5-10,13-15] have reported comparable ethnic differences to those observed in this study. As such they can probably be considered analogous to sex-specific differences.

The practical application of ethnic-specific standards require further "on-site" examination, but our results provide a useful analytic comparison tool for future research and should be considered when making clinical judgements about fetal surveillance, induction of labour, and postnatal nutrition.

As in all studies based on birth and death registrations, errors can occur in the estimation of gestational age, the recording or computer entry of birth weight or registration, and the linkage of live births and infant deaths. The deterministic linkage method used in British Columbia should minimize the latter type of error, and detailed analysis of the quality of the birth weight and gestational age data from this file have been reported [18]. Nonetheless, a few errors at very preterm gestations can have relatively large impacts on birth weight for gestational age and survival at those gestations. Moreover, small numbers of events (perinatal deaths and SGA live births) at very preterm gestations lead to statistical instability (bumps, peaks, and valleys) in trends for those events in the smaller ethnic groups (Chinese, South Asians, and First Nations) under study. Despite these limitations, we believe our results support the case for ethnic-specific standards for defining SGA.

## Conclusion

The concordance of perinatal mortality and SGA rates when based on ethnic-specific standards, and their discordance when based on a single standard, strongly suggests that the observed ethnic differences in fetal growth are physiologic, rather than pathologic, and make a strong case for ethnic-specific standards.

## Competing interests

The author(s) declare that they have no competing interests.

## Authors' contributions

WJK conceived the study, designed and performed all analyses (except Table 2), and drafted the manuscript. KSJ conceived the "fetuses at risk" analysis and carried out the analysis for Table 2. MSK directed the methods and analyses and drafted the manuscript. All authors participated in the study plan, reviewed all analyses, and edited the manuscript.

## Pre-publication history

The pre-publication history for this paper can be accessed here:


